# Effect of anti-androgens on body composition in transgender people: Secondary analysis of a randomized clinical trial

**DOI:** 10.1080/26895269.2025.2531421

**Published:** 2025-07-12

**Authors:** Lachlan M. Angus, Shalem Y. Leemaqz, James C.G. Doery, Ada S. Cheung

**Affiliations:** a Department of Medicine, The University of Melbourne, Victoria, Australia; b Department of Endocrinology, Austin Health, Victoria, Australia; c College of Medicine and Public Health, Flinders University, Adelaide, Australia; d Department of Chemical Pathology, Monash Health, Victoria, Australia; e Department of Medicine, Monash University, Clayton, Australia

**Keywords:** Anti-androgen, body composition, feminization, testosterone, transgender

## Abstract

**Background:**

Transgender people desiring feminization commonly use cyproterone acetate or spironolactone, but the optimal anti-androgen to achieve feminization of body composition is unclear.

**Aims:**

To assess the effect of anti-androgens on body composition. We hypothesized cyproterone acetate would result in a greater increase in body fat than spironolactone due to greater suppression of serum testosterone concentration and more potent androgen receptor antagonism.

**Methods:**

Secondary analysis of a randomized clinical trial of transgender people aged 18+ years newly commencing estradiol and randomized to spironolactone 100 mg daily or cyproterone acetate 12.5 mg daily for six months. Pre-specified secondary outcomes including waist circumference, hip circumference, waist/hip ratio and whole body dual x-ray absorptiometry (DXA)

**Results:**

Sixty-three people were included in intention-to-treat analysis (cyproterone acetate *n* = 32, spironolactone *n* = 31). Overall, hip circumference increased but there was no change in waist circumference or waist/hip ratio. On whole body DXA, total body fat, percentage body fat, gynoid fat and android fat increased and trunk/limb fat ratio, android/gynoid fat ratio, total lean mass and appendicular lean mass decreased. No changes in visceral adipose tissue. Cyproterone acetate was associated with a greater increase in percentage total body fat (+1.98%, 0.34 to 3.63, *p* = 0.02), gynoid fat (+2.51%, 0.69 to 4.34, p = <0.01) and android fat (+2.63%, 0.40 to 4.85, *p* = 0.02) than spironolactone. Serum estradiol concentration was higher in the cyproterone acetate group (mean difference +228.38 pmol/L, 62.54 to 394.31, *p* < 0.01) but adjusted modeling remained statistically significant.

**Discussion:**

Feminization of body composition was evident following six months of hormone therapy. Cyproterone acetate caused a greater increase in percentage total body fat, gynoid fat and android fat compared to spironolactone. Given the change in the android/gynoid fat ratio was similar, and that the waist/hip ratio did not change over time, it is unclear whether cyproterone acetate offers an advantage to spironolactone.

## Introduction

Anti-androgens are commonly added to estradiol in transgender people seeking feminization to induce physical changes such as breast development, body fat redistribution and decreased facial and body hair (Cheung et al., 2019; Dekker et al., 2016; Hembree et al., 2017). Spironolactone and cyproterone acetate are commonly used as anti-androgens but differ in mechanisms of action: spironolactone is a moderate androgen receptor antagonist (IC50 77 nmol/L) (Bonne & Raynaud, 1974; Fagart et al., 2010) and weakly inhibits 17α-hydroxylase/17,20 lyase which is involved in testosterone biosynthesis (Kossor et al., 1991) while cyproterone acetate is a potent progestogen which inhibits testosterone synthesis *via* negative feedback on the hypothalamic pituitary testicular axis in addition to strong androgen receptor antagonism (IC50 7.1 nmol/L) (Neumann, 1996; Sonneveld et al., 2005). While a systematic review concluded that cyproterone acetate caused greater suppression of serum total testosterone concentrations than spironolactone, it’s not known whether this results in any clinically meaningful changes in feminization given the importance of androgen receptor antagonism (Angus et al., 2021).

Sexual dimorphism of body composition becomes apparent following puberty. Compared to cisgender men, cisgender women typically have a higher hip circumference, lower waist circumference and lower waist/hip ratio, and on whole body dual x-ray absorptiometry (DXA) have higher gynoid and appendicular fat, and lower android and truncal fat (Taylor et al., 2010). Observations in transgender people using feminizing hormone therapy resemble typical female pubertal changes with a relative increase in hip circumference, decrease in waist/hip ratio, and an increase in total body and gynoid fat, and a decrease in lean mass when assessed *via* whole body DXA (Gava et al., 2020; Klaver et al., 2018).

In the absence of robust evidence on comparative efficacy of anti-androgens, prescribing practices vary significantly worldwide reflecting local differences in cost, availability and experience. In this study of transgender people commencing estradiol, we aimed to assess the effects of cyproterone acetate and spironolactone on body composition, as feminization of body composition and shape is a commonly desired goal of therapy. We hypothesized that use of cyproterone acetate would result in a greater increase in total body fat and gynoid fat than spironolactone due to greater suppression of serum total testosterone and more potent antagonism of the androgen receptor.

## Materials and methods

This study was reported using the CONSORT 2010 statement: updated guidelines for reporting parallel group randomized trials (Schulz et al., 2010).

### Trial design

This was an investigator-initiated, double-blind, six-month randomized trial conducted at a single tertiary referral hospital in Melbourne, Australia. Ethics approval was obtained from Austin Health Human Research Ethics Committee (HREC/44503/Austin-2018) and LGBTIQ+ community-controlled organization Thorne Harbor Health’s Community Research Endorsement Panel (THH/CREP 20-002). The trial was prospectively registered on the Australian New Zealand Clinical Trials Registry (ACTRN12620000339954). The authors confirm that research complies with the Declaration of Helsinki and that participants provided written informed consent.

### Participants

Transgender people aged over 18 years newly commencing estradiol were eligible for enrollment. Participants were recruited from the Austin Health Gender Clinic, and local primary care and endocrine clinics.

Exclusion criteria included commencement of estradiol >6 wk prior to enrollment, preexisting hypogonadism (serum total testosterone concentration <10 nmol/L prior to commencing estradiol), previous treatment with anti-androgens, contraindications to treatment with spironolactone or cyproterone acetate and planned orchidectomy during the study period.

### Interventions and trial procedures

Participants were randomized to treatment with oral cyproterone acetate 12.5 mg daily or oral spironolactone 100 mg daily for six months, with administration recommended in the morning. Medications were prepared as identical capsules in appearance and taste and provided to participants at 0 and 3 months. In the absence of dose-finding studies with clinically meaningful outcomes to determine the optimal dose, the doses of spironolactone and cyproterone acetate used were derived from the median doses used in a cohort of transgender people, reflecting real world clinical practice in Australia (Angus et al., 2019).

Standardized estradiol therapy was commenced with oral estradiol valerate 4 mg daily or transdermal dose equivalent based on participant and clinician preference. Estradiol doses were subsequently titrated at months 1, 2 and 3 to achieve a serum estradiol concentration of 250–600 pmol/L to a maximum dose of oral estradiol valerate 8 mg or transdermal dose equivalent, in line with Australian consensus guidelines (Cheung et al., 2019).

### Outcomes

The following were pre-specified secondary outcomes:

*Whole body DXA*

Body composition was assessed by whole body DXA at 0 and 6 months using the Hologic Horizon DXA version 13.6.0.5 (Hologic Inc., Marlborough, MA, USA). All participants were scanned using the same machine by an experienced technician. Standard reports include computerized analysis of fat, lean and bone mineral content by body region (head, arms, trunk, legs, android, gynoid) and derived adipose mass measures (total body fat percentage, fat mass index, android/gynoid fat ratio, trunk/limb fat mass ratio, estimated visceral adipose tissue mass, volume and area) and lean mass measures (lean mass index, appendicular lean mass index). Results can be compared to normative population data from 2008 available from the National Health and Nutritional Examination Survey (NHANES) database for diagnostic purposes on an individual level (Kelly et al., 2009). Fat mass, lean mass and appendicular lean mass scale to height raised to the power of 2, i.e., height^2^, hence these values are adjusted for height and allow inter-individual comparison (Heymsfield et al., 2011).

*Waist circumference, hip circumference, waist hip ratio*

Waist circumference and hip circumference were measured at 0, 3 and 6 months. Waist circumference was measured at the smallest abdominal circumference and hip circumference was measured at the level of the greater trochanter. Waist/hip ratio was calculated by dividing the waist circumference by the hip circumference.

### Sample size

This is a secondary analysis of a randomized controlled trial in which the sample size calculation was based on the percentage of participants that suppressed serum total testosterone <2 nmol/L in a previously published cross-sectional study (Angus et al., 2019). To detect a difference of 40%, a sample size of 25 per group was required to achieve power 0.9 and level of significance 0.05. Using a conservative dropout of rate of 20%, we estimated that 64 participants would need to be recruited. A post-hoc power calculation for this secondary analysis indicated that a power of 81% was achieved to detect a treatment by time interaction effect size of 2.14 in percentage total body fat, within a linear mixed effects model including treatment group, time, treatment by time interaction, and three covariates.

### Randomization

Randomization was performed by an independent trials pharmacist with participants allocated in a 1:1 ratio according to a computer-generated randomization procedure, stratified in blocks of two for baseline age (<27 or ≥27 years) and body mass index (BMI) (<24.7 or ≥24.7 kg/m^2^). The age and BMI cutoffs to define groups and medication doses were based on median values from previous analysis of our clinic data (Gava et al., 2020). Age and BMI were incorporated into stratification given the potential confounding effect on feminization observed and a desire to have these variables evenly distributed between groups given our modest sample size (Heymsfield et al., 2011).

### Blinding

Participants, care providers and investigators were blinded to treatment allocation until data collection and data analysis was complete.

### Statistical methods

Statistical analysis was performed using R version 4.3.2 (R Foundation for Statistical Computing, Vienna, Austria) on an intention-to-treat and per protocol basis (R Core Team, 2022). Normally distributed continuous data were reported with mean and standard deviation (SD), and median and interquartile range (IQR) were reported for non-normally distributed data. Frequency and percentage were reported for categorical data. A linear mixed effects model was fitted for body composition outcomes over time, with treatment group, time, and treatment by time interaction term. Group differences at each timepoint were compared using pre-specified post-hoc contrasts adjusted for multiple comparisons. The main outcomes were reported as mean difference and corresponding 95% confidence intervals. Where there is no strong evidence of treatment by time interaction according to global test, an overall mean difference was reported. Two-sided P values <0.05 were deemed statistically significant.

## Results

### Participant flow

Participant flow for this clinical trial has been previously described in detail (Angus et al., 2024). Ultimately, 63 participants were included in intention-to-treat analysis (cyproterone acetate group *n* = 32, spironolactone group *n* = 31). Fifty-five participants completed the study per protocol (cyproterone acetate group *n* = 28, spironolactone group *n* = 27), reflecting a study completion rate of 87%.

#### Recruitment

Recruitment occurred between August 2020 and March 2022 and was stopped once the target of 25 participants completing the study per group had been achieved. Data collection was completed in September 2022. There were minor disruptions related to the COVID-19 pandemic and associated restrictions in Melbourne, Australia.

#### Baseline characteristics

Baseline group characteristics including age, height, weight, BMI, race and country of birth were comparable between groups and are presented in Table 1. Few participants self-reported co-existing cardiometabolic conditions such as hypertension, dyslipidaemia, diabetes mellitus, metabolic dysfunction associated fatty liver disease or ischemic heart disease.

**Table 1.** Baseline participant characteristics.

	Cyproterone acetate	Spironolactone
N	32	31
Age – years	25.3 (22.0 − 29.0)	25.1 (21.9 − 27.8)
Height – cm	178.3 (7.4)	178.5 (6.1)
Weight – kg	84.9 (21.2)	85.0 (23.0)
BMI – kg/m^2^	25.7 (21.5 − 30.0)	24.6 (21.1 − 31.9)
Country of birth – n (%)		
Australia	27 (84.4)	27 (87.1)
Race – n (%)^#^		
White	31 (96.9)	27 (87.1)
Aboriginal and Torres Strait Islander	1 (3.1)	1 (3.2)
Asian	2 (6.3)	4 (12.9)
African	0 (0)	2 (6.5)
Co-existing physical health conditions		
Hypertension	0 (0)	1 (3.2)
Dyslipidaemia	0 (0)	1 (3.2)
Diabetes mellitus	1 (3.1)	0 (0)
Metabolic dysfunction associated fatty liver disease	1 (3.1)	0 (0)
Ischemic heart disease	0 (0)	0 (0)

Normally distributed data reported as mean (standard deviation). Non-normally distributed data reported as median (interquartile range). Abbreviations: BMI, body mass index;

^#^Race: totals do not add to 100% as participants could identify with ­multiple racial backgrounds

#### Outcomes

Key outcomes are presented in Table 2 and Figure 1.

**Figure 1.**
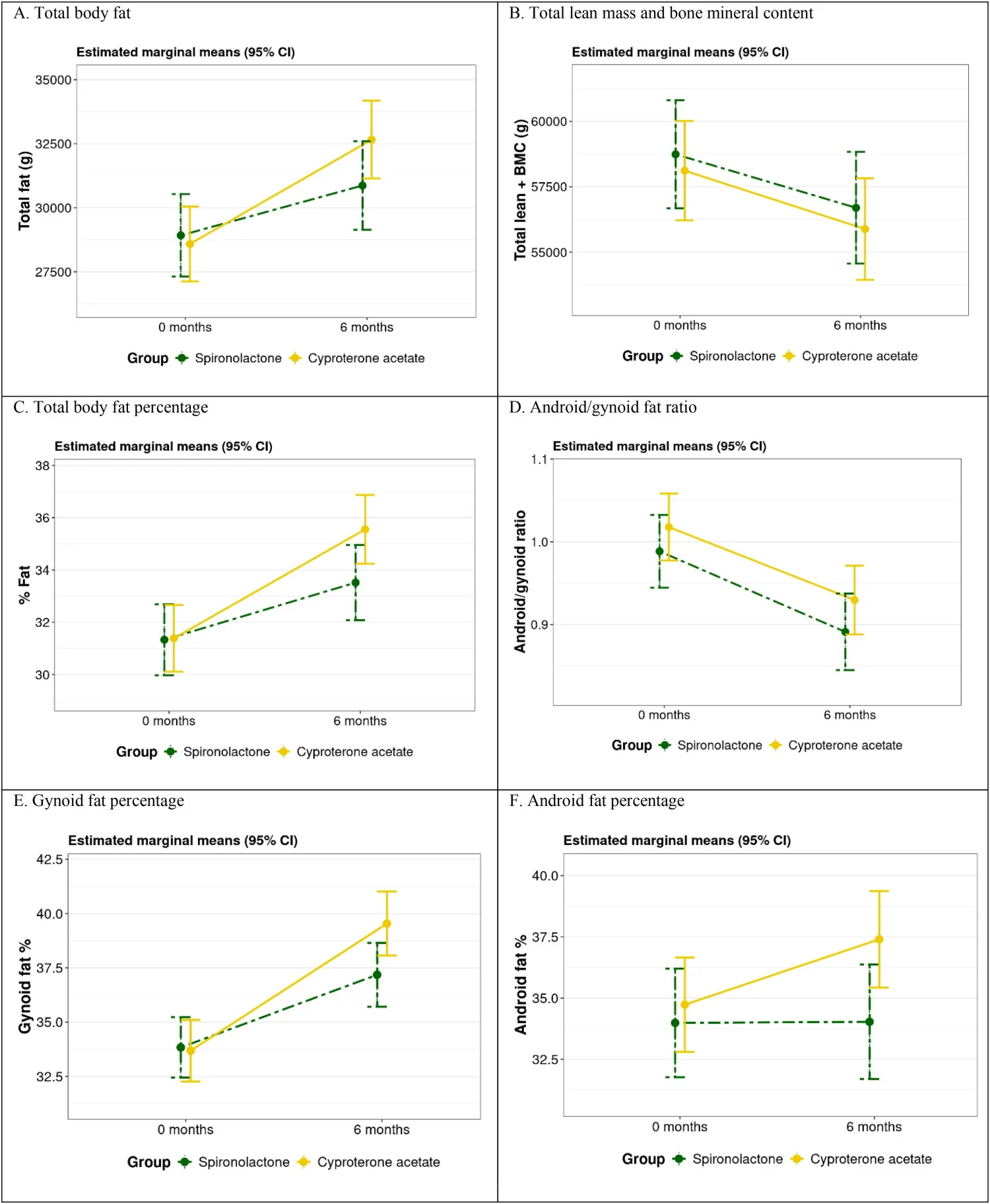
Plot of changes in selected body composition measures over time by treatment group. Between group differences were statistically significant for percentage total body fat, gynoid fat and android fat.

**Table 2.** Intention to treat analysis of key outcomes.

	Overall	Cyproterone acetate			Spironolactone			Between group comparison	
		Value	Mean difference (95% CI)	P	Value	Mean difference (95% CI)	P	Mean difference (95% CI)	P (interaction)
Anthropometry									
Waist circumference – cm									
0 months	89.3 (17.9)	89.9 (18.2)			88.7 (17.9)				
3 months	87. (17.2)	87.4 (16.8)	−0.77 (−2.36, 0.82)	0.3	87.4 (18.0)	−0.77 (−2.36, 0.82)	0.3		
6 months	93.1 (36.8)	88.9 (16.2)	−0.01 (−1.64, 1.63)	1.0	97.3 (49.5)	−0.01 (−1.64, 1.63)	1.0	−1.21 (−7.84, 5.42)	0.7
Hip circumference – cm									
0 months	98.7 (13.4)	99.3 (12.7)			98.1 (14.2)				
3 months	99.0 (11.8)	99.6 (11.6)	0.96 (−0.42, 2.34)	0.2	98.3 (12.1)	0.96 (−0.42, 2.34)	0.2		
6 months	100.5 (12.2)	101.1 (11.7)	1.61 (0.21, 3.01)	0.02	99.9 (12.8)	1.61 (0.21, 3.01)	0.02	1.36 (−0.69, 3.42)	0.2
Waist hip ratio									
0 months	0.9 (0.1)	0.9 (0.1)			0.9 (0.1)				
3 months	0.9 (0.1)	0.9 (0.1)	−0.02 (−0.04, −0.00)	0.05	0.9 (0.1)	−0.02 (−0.04, −0.00)	0.05		
6 months	0.9 (0.4)	0.9 (0.1)	−0.02 (−0.04, −0.00)	0.05	1.0 (0.5)	−0.02 (−0.04, −0.00)	0.05	−0.03 (−0.10. 0.04)	0.4
Whole body DXA									
Total body fat – kg									
0 months	29.41 (14.12)	28.27 (13.75)			30.69 (14.71)				
6 months	30.94 (13.54)	30.66 (13.64)	4.07 (2.66, 5.49)	<0.01	31.27 (13.76)	1.95 (0.28, 3.61)	0.02	2.13 (−0.06, 4.31)	0.06
Total lean mass and bone mineral content – kg									
0 months	58.62 (9.13)	57.82 (8.29)			59.53 (10.09)				
6 months	56.60 (8.02)	55.67 (7.24)	−2.16 (−2.95, −1.37)	<0.01	57.77 (8.95)	−2.16 (−2.95, −1.37)	<0.01	−0.71 (−3.43, 2.02)	0.6
Total body mass – kg									
0 months	78.77 (34.34)	77.48 (32.64)			80.19 (36.70)				
6 months	75.75 (35.57)	79.93 (29.86)	−1.30 (−11.97, 9.38)	0.8	71.23 (41.00)	−1.30 (−11.97, 9.38)	0.8	7.20 (−4.75, 19.15)	0.2
Lean mass index – kg/m^2^									
0 months	17.72 (2.54)	17.57 (2.32)			17.89 (2.81)				
6 months	16.91 (2.39)	16.74 (2.06)	−0.65 (−0.89, −0.42)	<0.01	17.14 (2.78)	−0.65 (−0.89, −0.42)	<0.01	0.02 (−0.47, 0.50)	0.9
Appendicular lean mass index – kg/m^2^									
0 months	7.81 (1.24)	7.71 (1.01)			7.93 (1.46)				
6 months	7.38 (1.15)	7.31 (0.98)	−0.36 (−0.49, −0.24)	<0.01	7.47 (1.36)	−0.36 (−0.49, −0.24)	<0.01	−0.05 (−0.35, 0.25)	0.7
Fat mass index – kg/m^2^									
0 months	9.27 (4.43)	8.98 (4.46)			9.60 (4.46)				
6 months	9.71 (4.32)	9.67 (4.35)	0.96 (0.60, 1.32)	<0.01	9.76 (4.40)	0.96 (0.60, 1.32)	<0.01	0.30 (−0.21, 0.80)	0.2
Total body fat – %									
0 months	31.72 (7.77)	31.22 (8.19)			32.28 (7.39)				
6 months	33.93 (7.25)	34.12 (7.23)	4.17 (3.06, 5.28)	<0.01	33.69 (7.45)	2.19 (0.98, 3.39)	<0.01	1.98 (0.34, 3.63)	0.02
Android fat – kg									
0 months	2.62 (1.61)	2.53 (1.60)			2.73 (1.65)				
6 months	2.46 (1.50)	2.49 (1.48)	0.10 (−0.02, 0.21)	0.1	2.43 (1.55)	0.10 (−0.02, 0.21)	0.1	0.14 (−0.15, 0.42)	0.3
Android mass – kg									
0 months	6.13 (3.11)	5.97 (2.98)			6.31 (3.29)				
6 months	5.63 (3.02)	5.89 (2.68)	−0.34 (−1.16, 0.48)	0.4	5.36 (3.39)	−0.34 (−1.16, 0.48)	0.4	0.58 (−0.36, 1.51)	0.2
Android fat – %									
0 months	34.95 (10.59)	34.70 (11.21)			35.23 (10.08)				
6 months	34.77 (10.21)	35.15 (10.46)	2.67 (1.44, 3.90)	<0.01	34.30 (10.14)	0.04 (−1.81, 1.90)	1.0	2.63 (0.40, 4.85)	0.02
Gynoid fat – kg									
0 months	5.03 (2.23)	4.83 (2.19)			5.23 (2.31)				
6 months	5.58 (2.01)	5.59 (1.95)	0.95 (0.65, 1.26)	<0.01	5.57 (2.13)	0.56 (0.33, 0.80)	<0.01	0.39 (0.00, 0.77)	0.05
Gynoid mass – kg									
0 months	12.70 (5.68)	12.46 (5.43)			12.97 (6.03)				
6 months	12.42 (5.81)	13.12 (4.84)	0.00 (−1.75, 1.76)	1.0	11.66 (6.72)	0.00 (−1.75, 1.76)	1.0	1.12 (−0.83, 3.07)	0.3
Gynoid fat – %									
0 months	33.98 (6.47)	33.41 (6.84)			34.62 (6.09)				
6 months	37.91 (5.82)	38.54 (5.31)	5.86 (4.48, 7.23)	<0.01	37.12 (6.45)	3.35 (2.14, 4.55)	<0.01	2.51 (0.69, 4.34)	<0.01
Android/Gynoid fat ratio									
0 months	1.01 (0.16)	1.02 (0.17)			1.00 (0.15)				
6 months	0.90 (0.15)	0.90 (0.16)	−0.09 (−0.11, −0.07)	<0.01	(0.16)	−0.09 (−0.11, −0.07)	<0.01	0.03 (−0.02, 0.09)	0.2
Trunk/limb fat mass ratio									
0 months	1.03 (0.18)	1.07 (0.19)			0.99 (0.16)				
6 months	0.91 (0.17)	0.90 (0.16)	−0.11 (−0.14, −0.09)	<0.01	0.92 (0.19)	−0.11 (−0.14, −0.09)	<0.01	0.08 (0.00, 0.16)	0.05
Trunk/limb fat % ratio									
0 months	0.93 (0.12)	0.95 (0.12)			0.91 (0.11)				
6 months	0.85 (0.12)	0.84 (0.12)	−0.08 (−0.09, −0.06)	<0.01	0.85 (0.13)	−0.08 (−0.09, −0.06)	<0.01	0.04 (−0.01, 0.09)	0.1
Estimated VAT mass – g									
0 months	527.65 (286.08)	524.85 (274.74)			530.79 (304.25)				
6 months	480.78 (262.65)	463.04 (241.84)	−1.98 (−33.68, 29.72)	0.9	502.95 (291.45)	−1.98 (−33.68, 29.72)	0.9	30.63 (−56.70, 117.95)	0.5
Estimated VAT area – cm^2^									
0 months	109.47 (59.41)	08.86 (57.04)			110.16 (63.19)				
6 months	99.67 (54.44)	95.96 (50.08)	−0.53 (−7.15, 6.08)	0.9	104.30 (60.45)	−0.53 (−7.15, 6.08)	0.9	6.28 (−11.87, 24.42)	0.5
Biochemistry									
Total testosterone – nmol/L									
0 months	17.17 (7.73)	17.30 (7.53)			17.02 (8.10)				
3 months	3.78 (5.52)	0.81 (0.40)	−16.59 (−18.58, −14.60)	<0.01	6.75 (6.60)	−10.40 (−13.38, −7.41)	<0.01		
6 months	2.87 (4.71)	1.48 (3.45)	−15.76 (−17.83, −13.69)	<0.01	4.15 (5.38)	−12.43 (−15.44, −9.42)	<0.01	−3.33 (−6.99, 0.32)	0.07
Sex hormone binding globulin – nmol/L									
0 months	30.58 (17.68)	27.42 (15.26)			34.35 (19.84)				
3 months	45.85 (31.13)	42.07 (28.73)	14.82 (9.93, 19.70)	<0.01	49.63 (33.48)	14.82 (9.93, 19.70)	<0.01		
6 months	46.37 (30.64)	44.76 (27.13)	15.45 (10.42, 20.48)	<0.01	48.04 (34.43)	15.45 (10.42, 20.48)	<0.01	−6.23 (−17.60, 5.13)	0.7
Estradiol– pmol/L									
0 months	183.58 (156.94)	152.03 (86.42)			218.50 (205.44)				
3 months	344.08 (167.81)	340.36 (181.61)	187.84 (79.51, 296.17)	<0.01	347.97 (154.99)	128.72 (7.23, 250.20)	0.04		
6 months	435.16 (290.26)	515.93 (351.19)	364.45 (252.57, 476.32)	<0.01	354.38 (186.99)	136.07 (13.53, 258.60)	0.03	228.38 (62.54, 394.31)	<0.01
Creatinine – μmol/L									
0 months	78.21 (10.60)	76.28 (10.44)			80.27 (10.57)				
3 months	74.19 (10.04)	70.90 (9.28)	−4.90 (−6.76, −3.03)	<0.01	77.61 (9.79)	−4.90 (−6.76, −3.03)	<0.01		
6 months	72.04 (11.78)	68.57 (8.92)	−6.94 (−8.82, −5.05)	<0.01	75.63 (13.38)	−6.94 (−8.82, −5.05)	<0.01	−6.78 (−11.72, −1.83)	<0.01

Values are reported as mean (standard deviation) unless otherwise stated. P refers to comparison from 0 to 3 months from 0 to 6 months within each group. The mean difference and corresponding 95% confidence interval and P (interaction) values refer to the time by treatment interaction estimated from the linear mixed effects model.

*Whole body DXA*

Overall, at six months there was a significant increase in body fat measures (total body fat, fat mass index, body fat percentage, gynoid fat mass and percentage) and a decrease in android/gynoid ratio, trunk/limb fat mass and lean mass indices (total lean mass and bone mineral content, lean mass index, appendicular lean mass index). There was no significant change to total body mass, estimated visceral adipose tissue mass or area.

Cyproterone acetate was associated with a greater increase in fat measures including total body fat percentage (mean difference +1.98%, 0.34 to 3.63, p = 0.02), gynoid fat percentage (+2.51%, 0.69 to 4.32, p < 0.01) and android fat percentage (+2.63%, 0.40 to 4.85, p = 0.02) than spironolactone, but no between-group difference was observed in total body fat, fat mass index, android fat mass, gynoid fat mass or android/gynoid ratio. There were no between-group differences in lean mass measures (lean mass index, appendicular lean mass index) or visceral fat measures (estimated mass and area). Further sensitivity analyses adjusting for differences in serum estradiol concentration between groups did not alter the statistical significance of these observed differences between groups.

*Clinical anthropometry*

Overall, there was a significant increase in hip circumference from 98.7 (13.4) cm at baseline to 100.5 (12.2) cm at six months (mean difference 1.61cm, 0.21 to 3.01, p = 0.02). However, there was no significant between group difference (mean difference 1.36cm, -0.69 to 3.42, p = 0.2). There was no significant change in waist circumference or waist hip ratio in either treatment group during the observed period.

*Biochemistry*

Over six months, there was a significant decrease in serum testosterone concentration from 17.17 (7.74) nmol/L at baseline to 2.87 (4.71) nmol/L at six months, with no between group difference (mean difference −3.33 nmol/L, −6.99 to 0.32, *p* = 0.07). There was a significant increase in serum estradiol concentration from 183.58 (156.94) pmol/L at baseline to 435.16 (290.26) pmol/L at six months, with significant between-group difference (mean difference 228.38, 62.45 to 394.31, *p* < 0.01), higher in the cyproterone acetate group. The serum creatinine concentration decreased from 78.21 (10.60) μmol/L at baseline to 72.04 (11.78) μmol/L at six months, with significant between group difference (mean difference −6.78, −11.72 to 1.83, *p* < 0.01), lower in the cyproterone acetate group.

## Discussion

In this secondary analysis of a randomized clinical trial in transgender people commencing estradiol, feminization of body composition was evident by six months of hormone therapy, with an observed increase in hip circumference, total fat mass and gynoid fat, decrease in trunk/limb fat ratio, android/gynoid fat ratio and total lean mass. There was no change in waist circumference, waist/hip ratio or estimated visceral adipose tissue mass. Cyproterone acetate use was associated with a greater increase in some fat measures (total body fat percentage, gynoid body fat percentage and android fat percentage) than spironolactone, despite similar serum total testosterone concentrations.

To date, there has been limited published data on the different clinical effects of feminizing hormone regimens, and instead a reliance on use of serum total testosterone concentration as a surrogate marker of efficacy. In terms of body composition changes, Gava et al. (2020) published 5-year data from a cohort of fifty transgender women treated with cyproterone acetate 50 mg daily or leuprolide acetate 3.75 mg monthly in addition to estradiol. This showed no significant change in waist circumference, hip circumference, waist/hip ratio, lean body mass or fat mass in either group. However, it should be noted that participants were treated with low doses of oral (1–2 mg/day) or transdermal estradiol and sample size may have affected statistical power. Recently Yaish et al. published the results of a prospective open-label non-randomized study where 22 hormone therapy naïve transgender women were treated with either oral estradiol 2 mg daily plus cyproterone acetate 10 mg daily or sublingual estradiol 2 mg daily for six months and body composition was assessed *via* clinical examination, whole body DXA and bioimpedance analysis (Yaish et al., 2025). Clear body composition changes were observed over six months, broadly similar to previously published literature, with an increase in fat mass and gynoid fat, a decrease in the android/gynoid fat ratio, waist/hip ratio and lean mass (Klaver et al., 2018; Yaish et al., 2025). While body composition changes were largely similar between groups, there was relative preservation of truncal lean mass in those treated with sublingual estradiol despite similar serum total testosterone concentrations to the oral estradiol and cyproterone acetate group (Yaish et al., 2025). This may support the notion that unmeasured androgen receptor antagonism with cyproterone acetate is an important factor contributing to observed changes. Notably, a small sample size may have limited the ability to detect between group differences. While we observed feminization of body composition over six months in our study, it is less clear whether the differences in percentage total body fat, gynoid fat and android fat are clinically significant given the waist hip ratio and android/gynoid fat ratio were similar.

Despite apparent increases in body fat and decreases in lean mass over the study period, there was no increase in estimated visceral adipose tissue area or mass. These findings support that of Klaver et al. (2022) who reported a slight decrease in visceral adipose tissue and in the visceral adipose tissue/total body fat ratio in a large cohort of transgender women treated with estradiol and cyproterone acetate for 12 months. Several studies have also shown a positive effect of estradiol and cyproterone acetate on lipid profiles with a decrease in total cholesterol, LDL-cholesterol and triglycerides albeit also a decrease in HDL-cholesterol (Klaver et al., 2022; van Velzen et al., 2019). This is relevant given some early studies of body compositional changes in transgender people observed increased visceral adipose tissue deposition with transverse magnetic resonance imaging (Elbers et al., 1999) and the known association between increased visceral adipose tissue and cardiovascular risk (Cesaro et al., 2023). We did not observe a significant change in android fat mass in either group, which has previously been associated with insulin resistance (Bretherton et al., 2021). However, we did note an greater increase in android fat percentage in the cyproterone acetate group than the spironolactone for which the clinical significance is uncertain. Unfortunately, we did not measure plasma glucose concentrations or insulin concentrations to ascertain any between-group differences in insulin resistance. A recent meta-analysis concluded that there is an increased risk of cardiovascular disease among transgender women compared to cisgender men (van Zijverden et al., 2024). Interestingly, this may be independent of gender affirming hormone therapy (Karalexi et al., 2022) with other potential factors considered including the minority stress model (Frost & Meyer, 2023) and increased prevalence of psychiatric comorbidities, substance use and lower socioeconomic status of people in the transgender community compared to the general population (Karalexi et al., 2022).

Notably, participants had a baseline BMI comparable to previously published clinic data (Angus et al., 2019) and to general population data for Australian men aged 25–34 years old (median BMI 26.8 kg/m^2^) (Australian Bureau of Statistics, 2022). Despite this, participants had a relatively high mean total body fat percentage at baseline of 31.72% (7.77) compared to an age-matched white population average of 24.6% (6.49) (Australian Bureau of Statistics, 2018; Kelly et al., 2009). Conversely, participants had a relatively low mean lean mass index of 17.72 (2.54) kg/m^2^ compared to an age-matched white population of 19.31 (2.52) kg/m^2^. The factors contributing to this are likely complex and multifactorial including minority stress, decreased activity, poorer nutrition and disordered eating (Kirby & Linde, 2020). A recent review highlighted decreased self-reported activity and sports participation among transgender children and adolescents and barriers to participation including transphobia, exclusion or discrimination (Austin et al., 2024). Additionally, studies of nutrition in amongst trans people have identified themes of food insecurity, high fat intake, low intake of fruit, vegetables and grains and disordered eating behaviors including restriction, binge eating and purging (Kirby & Linde, 2020; Sousa & Amaral, 2024).

The differences in serum total testosterone concentration and estradiol concentration were discussed in detail in our previous publication (Angus et al., 2024). There was no between-group difference in serum total testosterone concentration at six months, but the odds of suppressing serum total testosterone concentration to <2 nmol/L was much more likely with cyproterone acetate, in keeping with existing literature and cyproterone acetate’s potent progestogenic suppression of testicular testosterone production (Angus et al., 2019). The difference in serum estradiol concentration was unexpected given both groups were treated with identical standardized estradiol regimens and titration protocols. However, this was not apparent at three months and may be explained by differences in estradiol formulations between groups and timing of administration relative to blood sampling. Further sensitivity analyses adjusting for differences in serum estradiol concentration were performed, however this did not alter the statistical significance of our findings. We noted a significantly higher serum creatinine concentration in the spironolactone group than the cyproterone acetate group, though it remained within the normal reference range. Given the decreases in lean mass measured were comparable between groups, we hypothesize that this may instead relate to spironolactone’s diuretic effects.

Strengths of this study include its prospective randomized controlled trial design, use of clinical endpoints and low drop-out rate of participants. Limitations include unknown optimal dosing of both cyproterone acetate and spironolactone for feminization, short intervention time of six months, difficulty comparing serum estradiol concentrations in a population using difference estradiol formulations, lack of information regarding diet and physical activity which may impact body composition changes, difficulty defining clinical significance in relation to observed differences in whole body DXA indices and limited generalizability of findings due to a predominantly young, white and comorbidity-free population.

## Conclusions

In this secondary analysis of a randomized clinical trial of transgender people commencing estradiol, we noted a significant increase in total body fat, total body fat percentage, gynoid fat percentage and android fat percentage, a decrease in total lean mass, appendicular lean mass, trunk/limb fat mass ratio, android/gynoid fat ratio and no change in estimated visceral adipose tissue mass. There was a significant increase in hip circumference but no change in waist circumference of waist hip ratio. Cyproterone acetate use was associated with a greater increase in some fat measures (total body fat, gynoid fat and android fat percentage) than spironolactone. Further research is needed to ascertain the clinical significance of these findings given there was no between-group difference in the android/gynoid fat ratio or waist/hip ratio.

## Data Availability

The data that support the findings of this study are available from the corresponding author, ASC, upon reasonable request.
